# Assessment of publication time in *Campbell Systematic Reviews*: A cross‐sectional survey

**DOI:** 10.1002/cl2.70011

**Published:** 2024-12-15

**Authors:** Bei Pan, Long Ge, Xiaoman Wang, Ning Ma, Zhipeng Wei, Lai Honghao, Liangying Hou, Kehu Yang

**Affiliations:** ^1^ Centre for Evidence‐Based Medicine, School of Basic Medical Science Lanzhou University Lanzhou Gansu China; ^2^ Centre for Evidence‐Based Social Science, School of Public Health Lanzhou University Lanzhou Gansu China

**Keywords:** Campbell, cross‐sectional, publication time, survey, systematic review

## Abstract

Delayed publication of systematic reviews increases the risk of presenting outdated data. To date, no studies have examined the time and review process from title registration and protocol publication to the final publication of Campbell systematic reviews. This study aims to examine the publication time from protocol to full review publication and the time gap between database searches and full review publication for Campbell systematic reviews. All Campbell systematic reviews in their first published version were included. We searched the Campbell systematic review journals on the Wiley Online Library website to identify all completed studies to date. We manually searched the table of contents of all Campbell systematic reviews to obtain the date of title registration from the journal's website. We used SPSS software to perform the statistical analysis. We used descriptive statistics to report publication times which were calculated stratified by characteristics, including year of review publication, type of reviews, number of authors, difference in authors between protocol and review, and Campbell Review Groups. Non‐normal distributed data were reported as medians, interquartile range, and range, and normal distributed data will be reported as mean ± standard deviation. And we also visualized the overall publication time and the distribution of data. Approximately 18% of reviews were published within one to 2 years, faster than the aims set by Campbell systematic review policies and guidelines, which was 2 years. However, more than 40% of the reviews were published more than 2 years after protocol publication. Furthermore, over 50% of included reviews were published with a time gap of more than 2 years after database searches. There was no significant difference between Campbell coordinating groups' median publication times and time gap from searches of databases to full review publication existed. However, the methods group only published one full review with almost a 3‐year time gap from searches of databases to review publication. And there was a major difference between specific types of review. Systematic reviews had the longest median publication time of 2.4 years, whereas evidence and gap maps had the lowest median publication time of 13 months. Half of Campbell reviews were published more than 2 years after protocol publication. Furthermore, the median time from protocol publication to review publication varied widely depending on the specific type of review.

## 1 PLAIN LANGUAGE SUMMARY

Half of Campbell's reviews were published more than 2 years after the last search date. Strategies for lowering the processing time are necessary.

### 1.1 The review in brief

Furthermore, systematic reviews take much more time than evidence and gap maps to publish.

### 1.2 What is this review about?

No previous studies have examined the time from title registration and publication of the protocol for a Campbell systematic review to the publication of the review itself, and the extent to which the Campbell reviews' publication time adheres to current guidelines, which was 2 years, is unclear. Delayed publication of systematic reviews increases the risk of outdated data.

### 1.3 What is the aim of this review?

This study aims to examine the publication time from protocol to full review publication and the time from searches of databases to full review publication for Campbell systematic reviews.

### 1.4 What studies are included?

This review identified 210 reviews from the Campbell systematic reviews journals on the Wiley Online Library website in May 2023. Of these, 186 were included in the analysis. More than 37.0% of reviews were published from 2019 to 2023 and were from the social welfare group. The vast majority were systematic reviews and only 6.1% were evidence and gap maps.

### 1.5 What are the main findings of this review?

About 18.0% of reviews were published faster than the aims of policies and guidelines of Campbell systematic reviews with a publication time between one and 2 years, however, more than 40% of the reviews were published more than 2 years after the protocol was published. And more than 50.0% of included reviews were published with more than 2 years' time gap after database searches. There was no significant difference between the 11 different Campbell coordinating groups' median publication times and time gap from searches of databases to full review publication. Systematic reviews had the longest median publication time of 2.4 years, whereas evidence and gap maps had the lowest median publication time of 13 months.

### 1.6 What do the findings of this review mean?

It is necessary to formulate a strategy to optimize the process of writing and publishing a Campbell review, and thus help lower publication times. This could reduce the risk of outdated evidence and the time commitment required for publishing a Campbell review. Improving timeliness of evidence is likely to be welcomed by people who wish to use Campbell reviews to inform decisions, and thus lead to more influence on policy and practice.

### 1.7 How up‐to‐date is this review?

This review included all the Campbell systematic reviews published to May 2023.

## 2 BACKGROUND

### 2.1 Description of the problem or issue

Systematic reviews compile the best available studies on a specific topic and synthesize the results of eligible studies, providing more valuable references for policymaking and practice than individual studies (Campbell Collaboration, 2018). Consequently, they are considered the highest level of evidence in evidence classification (Gates & March, 2016). In recent years, systematic reviews have gained popularity, with the number of annual publications increasing by more than 2000% since 1991 (Ioannidis, 2016).

The Campbell Collaboration (2020) supports the preparation and dissemination of high‐quality systematic reviews to investigate the effectiveness of social programs, policies, and practices. This enables policymakers, practitioners, and the public to make better‐informed decisions about policy interventions (Welch, 2018). There are 12 Campbell Review Groups that focus on specific topics and provide editorial and methodological support to assist authors in meeting the Campbell Collaboration's standards of methodology and reporting. Previous studies have shown that review protocols can reduce interim decision‐making and bias in the review process (Moher et al., 2015; Shamseer & Moher, 2015). Once a proposed title is approved and registered, authors in the Campbell Collaboration should submit a review protocol specifying the background, objectives, methods, a priori hypotheses, and statistical analysis for the final review. This is part of the rigorous methodology for producing a Campbell systematic review (The Campbell Collaboration, 2020). Campbell anticipates that developing a protocol would take 6 months, and writing the final review would take 18 months. The policies and guidelines for Campbell systematic reviews state that review teams should submit a draft protocol to the editor or managing editor of the sponsoring coordinating group no later than 1 year after title approval. Once the protocol is approved, review teams are advised to update the coordinating group editor or managing editor on their progress at least every 6 months and report any problems that may impede the timely delivery of the draft review (The Campbell Collaboration, 2020). However, the guidelines do not specify a time frame for submitting the full review. They only state that a protocol that has not resulted in a final published full review within 2 years can be withdrawn, and the review topic can then be made available to other interested review teams (The Campbell Collaboration, 2020). Despite these guidelines, the timeline from title registration to publication of the protocol for a Campbell systematic review, and then to the publication of the review itself, remains uncertain.

Similar to Campbell systematic reviews, Cochrane systematic reviews also adhere to high methodological standards and rigorous publication processes in medicine. Writing a Cochrane protocol typically takes 2–6 months, while completing a full review could take 1–2 years (Cochrane Community, 2021). Several studies have assessed the publication duration and process of Cochrane systematic reviews and have shown that complete Cochrane systematic reviews tend to take longer to publish than other systematic reviews (Andersen et al., 2020; Runjic et al., 2019; Tricco et al., 2008).

### 2.2 Why it is important to conduct this review?

To date, no studies have examined the time from title registration and protocol publication to the final publication of Campbell systematic reviews. The extent to which Campbell reviews' publication times adhere to current guidelines remains unclear. Delayed publication of systematic reviews increases the risk of presenting outdated data. Shojania et al. (2007) found that 7% of systematic reviews showed signs of being outdated at publication, and 11% showed signs of being outdated within 2 years of completing their systematic search. Wang et al. (2021) conducted a systematic review to assess the methodological and reporting quality of Campbell systematic reviews and found that the overall quality was high, especially after the introduction of the Methodological Expectations of Campbell Collaboration Intervention Reviews (MECCIR) in 2014. This high quality may be attributed to the stringent methodological standards that Campbell reviews must adhere to, which may result in longer publication times. Assessing the publication process and duration of Campbell systematic reviews will help devise strategies for optimizing and reducing the processing time from writing to publication without compromising quality. This could decrease the risk of outdated evidence and reduce the time commitment required for publishing a Campbell review.

## 3 OBJECTIVES

This study has three main objectives:

- To examine the time duration from the publication of a Campbell systematic review protocol to the publication of the completed Campbell systematic review.
- To describe publication times in relation to the characteristics of the reviews, including year of publication, type of review, number of authors, number of collaborative institutions, the time gap between the date of search completion and review publication, and the length and complexity of the included review (including the number of pages, tables, figures, studies included, analyses undertaken, and references).
- To describe the differences in publication times among Campbell Review Groups.

## 4 METHODOLOGY

### 4.1 Eligibility criteria

All Campbell systematic reviews in their first published version were included, provided they met the following criteria: (1) The review had both a published protocol and a subsequently published full‐text review; (2) Reviews from each of the following groups were included: business and management, children and young person's well‐being, climate solutions, crime and justice, disability, education, international development, knowledge translation and implementation, methods, and social welfare. We excluded reviews (1) if they are duplicates; (2) if they do not report these publication dates for title registration, protocol, or review; (3) The full review was not published after the protocol publication; and (4) if they have their protocol amended or updated before the full review was published.

### 4.2 Electronic searches

We searched the Campbell systematic review journals on the Wiley Online Library website to identify all completed studies to date. We manually searched the table of contents of all Campbell systematic reviews to obtain the date of title registration from the journal's website. However, we could not find title registration information for reviews published before 2019.

a) **Study screening and selection**
  Two reviewers (Ning Ma and Xiaoman Wang) independently screened all Campbell systematic reviews according to the eligibility criteria. Conflicts were resolved through discussion between the reviewers, with adjudication by a third reviewer (Bei Pan) when necessary.
b) **Data extraction**
  Two coders independently extracted data from all eligible studies. Any disagreements were resolved through discussion or by consulting a third party. A preliminary version of the coding scheme is presented in Table 1. We extracted the following information from the publicly available records of both reviews and protocols in Campbell systematic review journals: title registration dates, protocol publication dates, full review publication dates, types of review, review groups, review topics, number of authors, number of collaborative institutions, time gap between the date of search completion and review publication, and the length and complexity of the included review (including the number of pages, tables, figures, references, studies included, and analyses undertaken). The study sample size was determined by the available data from the Campbell Library that met the eligibility criteria.
c) **Data synthesis**
  We used SPSS software to perform the statistical analysis. We used descriptive statistics to report publication times, which were calculated and stratified by characteristics including year of review publication, type of review, number of authors, number of collaborative institutions, time gap between search completion and review publication, length and complexity of the included reviews (including number of pages, tables, figures, and references), and Campbell Review Groups. Non‐normally distributed data were reported as medians, interquartile ranges, and ranges, while normally distributed data were reported as means ± standard deviations. Additionally, we visualized the overall publication time and distribution of the data.

**Table 1:** Code information.

Category		Answer
Basic information	Title	Open answer
	Authors	Open answer
	Country	Open answer
	Number of Authors	Open answer
	Authors' Institutions	Open answer
Publication time	Title registration dates	Open answer
	Protocol publications dates	Open answer
	Full review publication dates	Open answer
Characteristics of the reviews	Campbell coordinating group	Aging
		Business and management
		children and young persons
		Climate solutions
		Crime and justice
		Disability
		Education
		International development
		Knowledge translation and implementation
		Methods
		Social Welfare
	Type of review	Systematic review
		Evidence and gap maps
		Methods research paper
	Topics of review	Open answer
	Time gap between the date the search was conducted and review publication	Open answer
	Number of collaborative institutions	Open answer
	Length and complexity of included review	Number of pages
		Number of tables
		Number of figures
		Number of references

### 4.3 Differences between protocol and review

In our original protocol, we planned to assess the time duration from title registration to publication of the protocol for Campbell systematic reviews. However, due to limited information on title registration for all included Campbell reviews, we did not analyze this data.

## 5 RESULTS

### 5.1 Reviews

**Figure 1:**
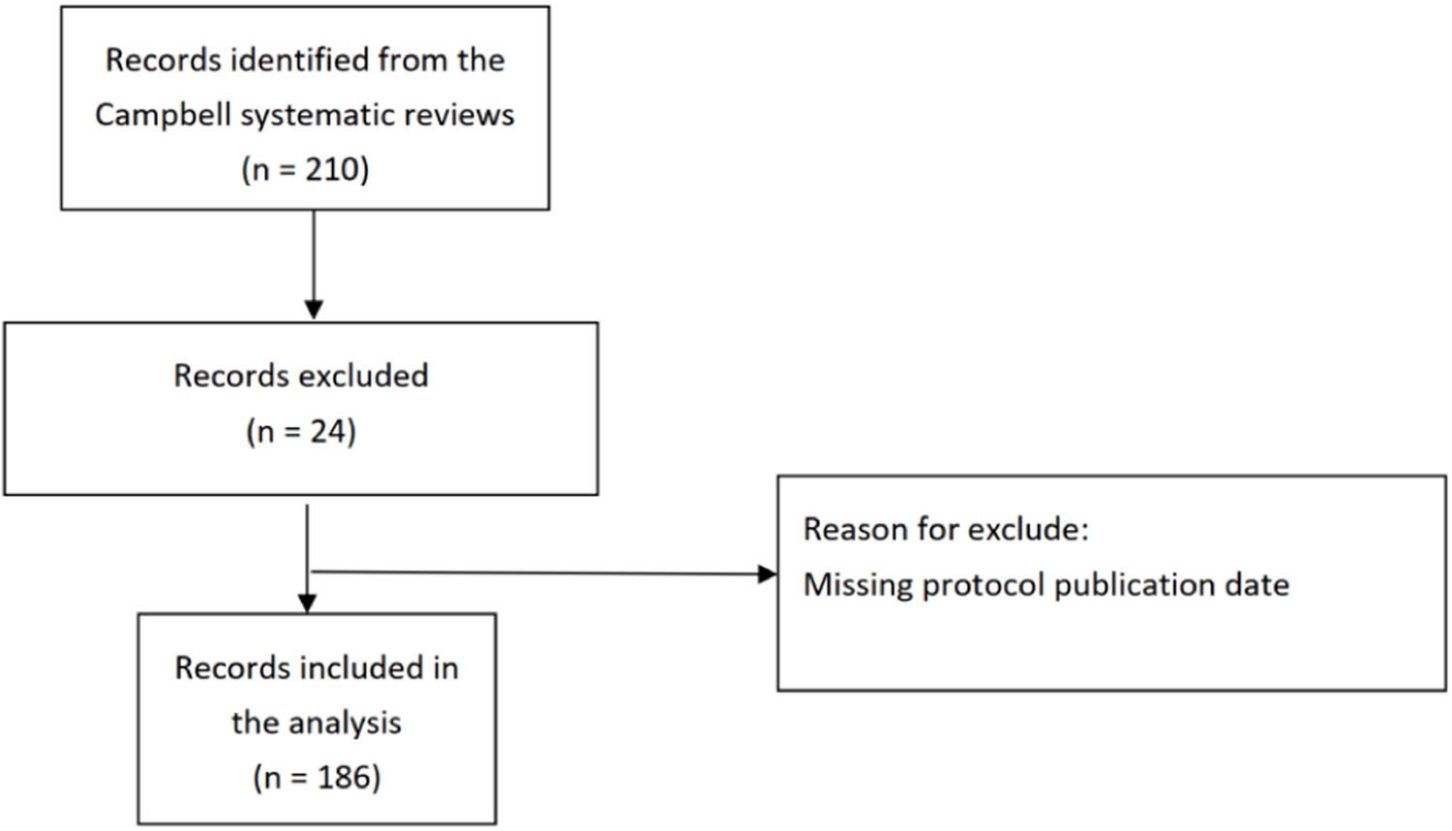
Flow diagram.

We identified a total of 210 reviews from the Campbell systematic review journals on the Wiley Online Library website in May 2023. Of these, 186 were included in the analysis. (Figure 1). The characteristics of included reviews and publication times for specific characteristics are presented in Table 2. Campbell reviews published from 2019 to 2023 account for 37.1% (*n* = 69) of all included reviews. More than 30% (*n* = 64) of reviews were from the social welfare group. The vast majority (92.5%, *n* = 172) were systematic reviews, 6.1% (*n* = 13) were evidence and gap maps, and only one study was a methods research paper. More than half (*n* = 56) of the Campbell reviews were written by one to three authors, while only 5.4% (*n* = 10) were written by more than seven authors. And 44.6% (*n* = 83) of all included reviews exceeded the final review deadline (18 months) set by the Campbell Collaboration (2020).

**Table 2:** Characteristics of the included Campbell reviews.

Characteristics	The Campbell Reviews		
	*n*	%	Median (range, months)
**Year of review publication**			
2003–2006	10	5.38	34 (0–60)
2007–2010	35	18.82	17 (0–66)
2011–2014	41	18.28	21 (6–100)
2015–2018	31	16.67	22 (10–69)
2019–2023	69	37.10	21 (1–130)
**Campbell coordinating group**			
Aging	0	0	0
Business and management	0	0	0
Children and young persons	1	0.54	9
Climate solutions	0	0	0
Crime and justice	50	26.88	21 (0–108)
Disability	3	1.61	21 (7–27)
Education	24	12.90	22 (6–100)
International development	43	23.12	21 (1–84)
Knowledge translation and implementation	0	0	0
Methods	1	0.54	18
Social Welfare	64	34.41	21 (0–130)
**Type of review**			
Systematic review	172	92.47	21 (0–130)
Evidence and gap maps	13	6.99	13 (4–36)
Methods research paper	1	0.54	18
**Number of collaborative institutions**			
1–3	137	73.66	21 (0–100)
4–6	39	20.97	21 (0–130)
>7	10	5.38	21 (9–84)
**Number of authors on the reviews**			
1–3	56	30.11	21 (0–100)
4–6	90	48.39	22 (0–130)
7–9	22	11.83	21 (8–69)
10–12	14	7.53	21 (4–84)
>12	3	1.61	21 (13–29)
**Number of pages**			
10–50	62	33.33	21 (0–100)
51–100	39	20.97	21 (2–130)
101–150	28	15.05	21 (0–60)
151–200	13	6.99	23 (8–84)
201–250	7	3.76	21 (12–31)
251–300	2	1.08	22
301–350	4	2.15	24 (14–30)
351–400	1	0.54	13
**Number of tables**			
0–5	39	20.97	29 (1–108)
6–10	59	31.72	26 (0–100)
11–15	33	17.74	19 (0–79)
15–20	5	2.69	30 (12–46)
>21	50	26.88	26 (0–130)
**Number of figures**			
0–5	74	39.78	21 (0–130)
6–10	36	19.35	23 (0–108)
11–15	30	16.13	21 (1–61)
15–20	17	9.14	21 (1–69)
>21	33	17.74	21 (5–84)
**Number of references**			
0–30	4	2.15	17 (6–66)
31–60	14	7.53	21 (0–50)
61–90	28	15.05	21 (0–57)
91–120	28	15.05	22 (8–100)
>120	112	60.22	26 (1–130)

### 5.2 Publication time

**Figure 2:**
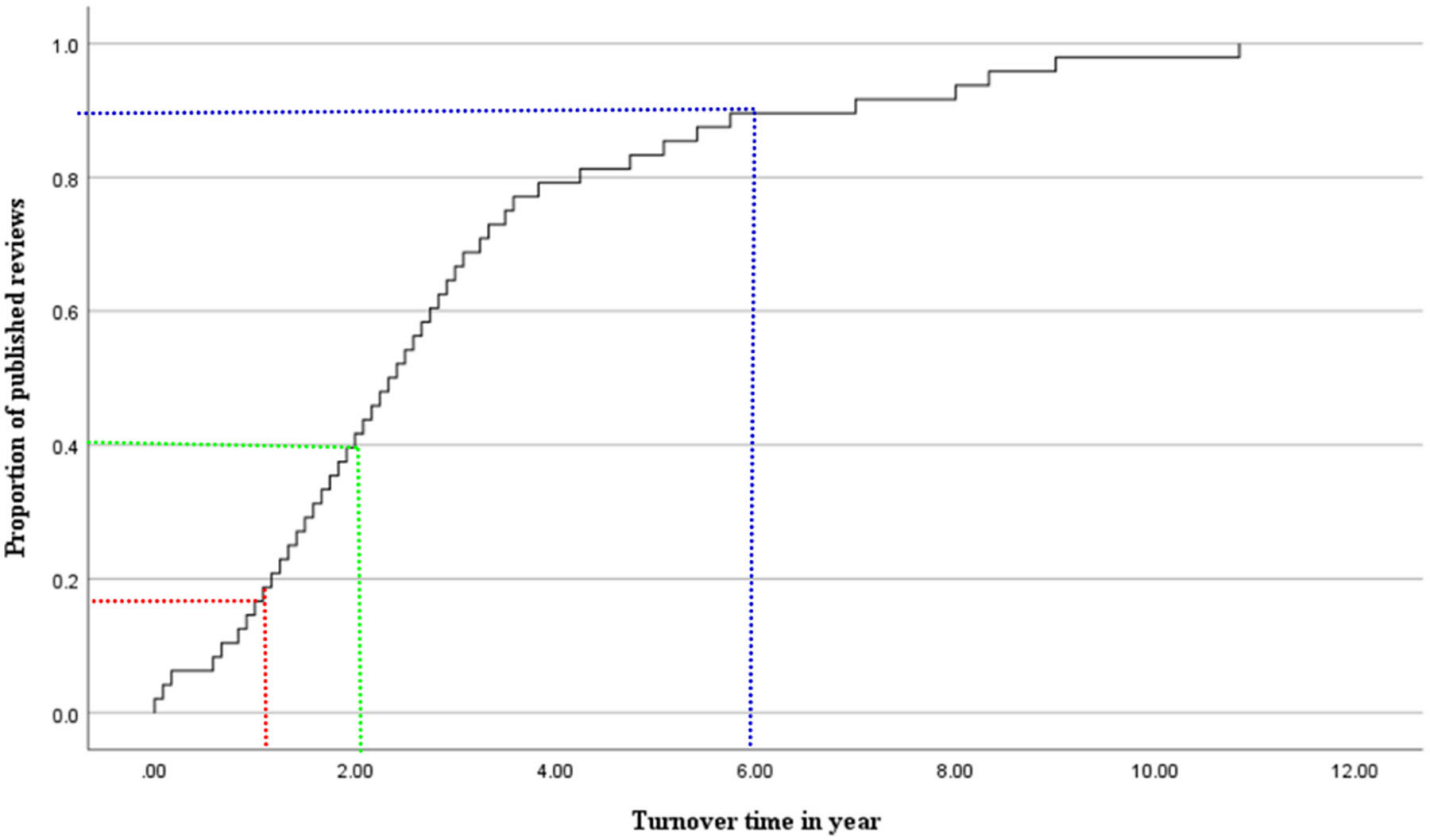
A Kaplan–Meier curve presenting the time from publication of the protocol to publication of the Campbell review. Red line: reviews published after 1 year. Green line: reviews published after 2 years. Blue line: reviews published after 5 years; *y*‐axis: the proportion of published reviews; *x*‐axis: a function of time from publication of protocols (the) measured in years. The maximum time range shown in the graph is 12 years.

**Figure 3:**
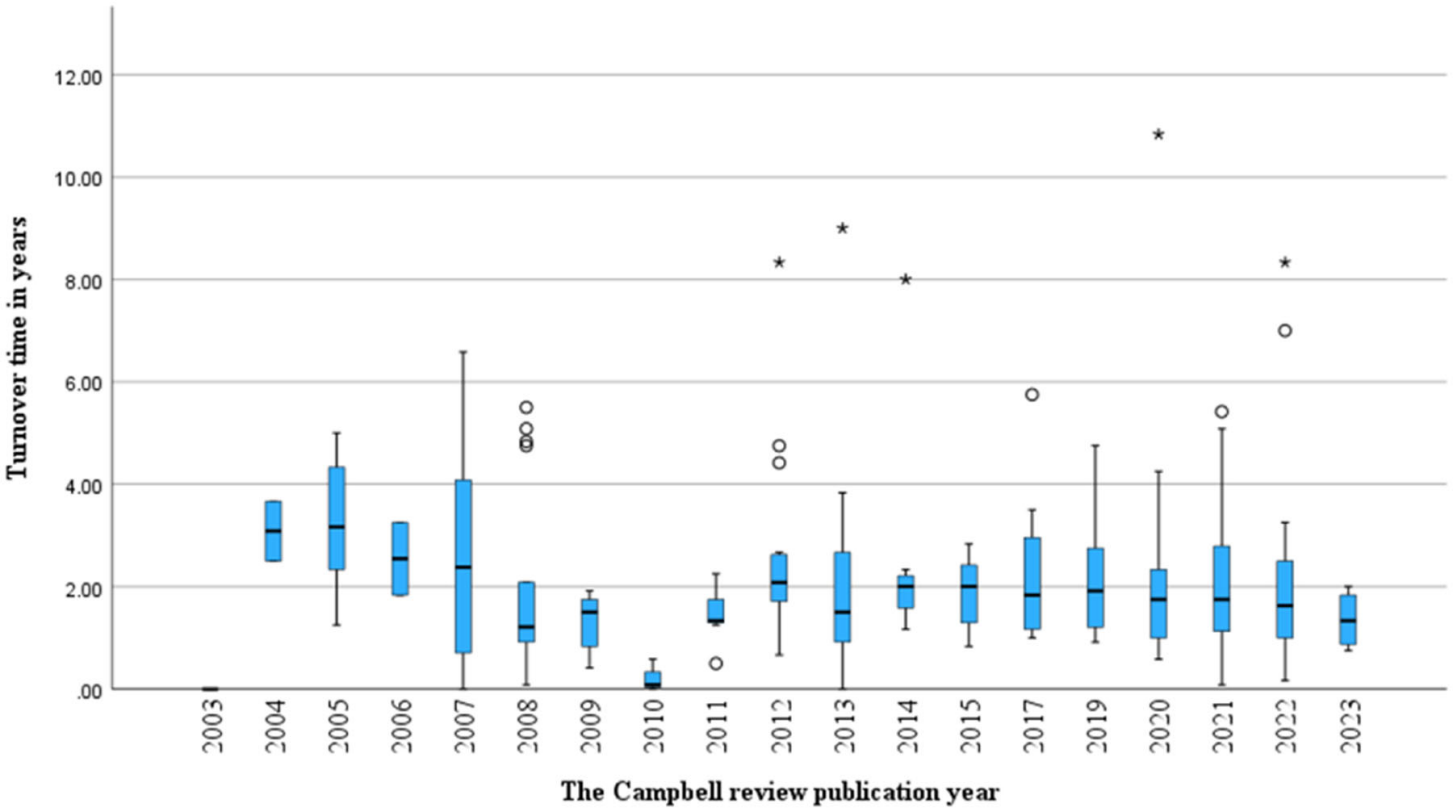
Boxplots illustrating the change in publication time throughout the years from 2003 to 2023 grouped by year of publication of review. no study published in 2018.

Figure 2 shows that the median publication time from protocol to full review publication for all included reviews was 2.4 years, with an interquartile range from 1.8 to 2.9 years. The median publication time for the 4‐year interval between 2003 and 2006 was 34 months, while the median publication time between 2007 and 2023 decreased to 17–21 months. When focusing on specific types of reviews, systematic reviews accounted for over 90% of included reviews and had a median publication time of 21 months, while evidence and gap maps had a relatively shorter median publication time of 13 months (Table 2). The median publication times grouped by Campbell coordinating groups were similar. For the number of authors and institutions, the median publication times were also similar. Regarding the number of pages, the highest median publication times were associated with reviews of 151–200 pages and 301–350 pages. For the number of tables, the highest median publication times were associated with 15–20 tables. For the number of figures, the highest median publication times were associated with 6–10 figures. For the number of references, the highest median publication times were associated with more than 120 references. Of all included reviews, about 18% were published less than 1 year after protocol publication (Figure 2). Of these, 3 reviews were published within 1 month after their protocol publication (excluding those published on the same day), while 5 were published on the same day as their protocol. Approximately 40% of Campbell reviews were published between 1 and 2 years after protocol publication. Figure 3 visualizes publication times for individual years.

The median time gap from database searches to full review publication was 1.67 years, with a full range of 0.17–7 years. The median time gaps from database searches to full review publication for specific Campbell coordinating groups were similar, except for the methods group, which showed the highest search gap time (3 years) with only one full review. Regarding specific types of reviews, systematic reviews were associated with longer search gap times. Of all analyzed reviews, 19% were published within 1–2 years after database searches. However, more than 50% of included reviews were published with a time gap of more than 2 years after database searches (Figure 2).

## 6 DISCUSSION

### 6.1 Main findings

In this study, we systematically investigated the publication time from protocol to full review publication and the time gap from database searches to full review publication. We found that about 18% of reviews were published faster than the aims set by Campbell systematic review policies and guidelines, with a publication time between one and 2 years. Of these, three reviews were published within 1 month after their protocol publication (excluding those published on the same day), while 5 were published on the same day as their protocol. Above reviews that are published very quickly after protocol or on the same day are likely to be reviews conducted outside the Campbell process initially, and then “Campbellised” to ensure they meet Campbell standards. However, more than 40% of the reviews were published more than 2 years after protocol publication. Furthermore, more than 50% of included reviews were published with a time gap of more than 2 years after database searches.

There were no significant differences in median publication times and time gaps from database searches to full review publication among Campbell coordinating groups. However, the methods group published only one full review, with a time gap of almost 3 years from database searches to review publication. There were major differences between specific types of reviews. Systematic reviews had the longest median publication time of 2.4 years, whereas evidence and gap maps had the lowest median publication time of 13 months.

### 6.2 Comparison with previous study

We systematically performed a literature search and found no relevant studies assessing the publication time of Campbell reviews. However, several studies have assessed the publication duration and process of Cochrane systematic reviews and have shown that complete Cochrane systematic reviews tend to take longer to publish than other systematic reviews (Andersen et al., 2020; Runjic et al., 2019; Tricco et al., 2008). The results from a recent study showed that the median publication time of Cochrane systematic reviews from 1995 to 2019 was 2 years, and Cochrane reviews seemed to be publishing more slowly than in previous years (Andersen et al., 2020). The median publication time from protocol to full review publication for all included Campbell reviews in our study was 2.4 years, which is similar to Cochrane reviews. This median publication time does not meet the policies and guidelines of Campbell systematic reviews, which specify a target of 18 months. For authors, the barrier may consist of large time commitments that may deter some from writing Campbell reviews. For decision‐makers, the barrier may be the long wait times for answers to clinical decision‐making questions that often require quick responses. Therefore, Campbell should develop methodologies to expedite the completion of reviews.

### 6.3 Strengths and limitations

This study has several strengths. First, it is the first to assess the entire corpus of Campbell systematic reviews from 2003 to May 2023. Second, we excluded updated protocols and reviews to provide more comparable data for analysis. Third, we followed the STROBE guideline reporting checklist to ensure reproducibility and transparency. Finally, the long period of included reviews could result in more reliable results.

This study also has some limitations. As of May 2023, only 186 full reviews were included in the analysis, which may not provide a vast amount of data for highly reliable results. We aimed to examine the time duration from title registration to publication of the protocol for Campbell systematic reviews, but information for most reviews registered before 2019 was not available. This analysis may be performed on a sample of Campbell reviews in future research. Another major limitation of this study is our failure to collect data on the breadth of each review. The breadth of a review, including its scope of studies, number of included studies, and complexity of research questions, may significantly impact completion times. The breadth of a review likely directly affects the workload in literature searching, screening, data extraction, and analysis. Broader or more complex reviews may require longer completion times, while narrower‐scope reviews might be completed more quickly.

While we cannot directly quantify the impact of review breadth on completion times, the observed variations in completion times may be partly attributed to this factor. According to field experts, review complexity and breadth are considered key factors influencing completion times (Borah et al., 2017). Interdisciplinary reviews or those involving multiple complex interventions often require more time to synthesize and analyze evidence. The lack of data on this important variable means our results may not fully explain all reasons for variations in completion times. This might have led to over‐ or underestimation of the influence of other factors. Future research could consider developing a standardized tool to assess the breadth and complexity of reviews, which would facilitate more accurate comparisons across studies.

While exploring factors influencing the completion time of Campbell systematic reviews, we recognize that the experience level of review teams is likely an important variable that we were unable to quantify in this study. Team experience can significantly affect the efficiency and quality of systematic reviews. More experienced teams may be more familiar with systematic review methodology and have more established workflows, potentially shortening completion times. However, due to data and resource constraints, this study was unable to directly measure and analyze the impact of team experience levels on completion times. This is a significant limitation of our research.

Additionally, we did not analyze the potential relationship between the scope of systematic reviews (such as the breadth of search strategies, number of included studies, etc.) and their publication times. Such an analysis could provide additional insights into factors affecting the publication times of systematic reviews. Future research should consider exploring the relationship between the scope of systematic reviews and their publication times. This could include analyzing the correlation between factors such as the number of databases searched, total studies screened, number of studies ultimately included, geographical scope of studies, and publication timelines. Such analysis might reveal whether more comprehensive reviews tend to have longer publication times, thus providing important information for improving the efficiency of systematic reviews.

### 6.4 Implications for policy, practice, and research

This study's findings have significant implications for policy, practice, and future research in Campbell systematic reviews. The Campbell Collaboration may need to revise its guidelines regarding timelines from protocol registration to full publication and implement strategies to reduce the time between database searches and publication. This could include optimizing project management, streamlining peer‐review processes, and utilizing technology to expedite various review stages. However, maintaining the high quality of Campbell reviews remains crucial while pursuing efficiency. Future research should focus on identifying factors contributing to publication delays, comparing Campbell review timelines with those of other systematic review organizations, and developing interventions to reduce publication time without compromising review quality. By addressing these issues, the Campbell Collaboration can optimize its review process, ensuring timely dissemination of high‐quality evidence to inform decision‐making in social sciences and related fields. This study reveals significant findings regarding the publication timelines of Campbell systematic reviews. Notably, half of Campbell reviews were published more than 2 years after their protocol registration, with substantial variations in median publication times across different review types. These results underscore the pressing need for optimizing the writing and publishing process of Campbell reviews. In conclusion, this study provides valuable insights into the current state of Campbell review publication timelines and offers a foundation for developing targeted strategies to optimize the review process. By addressing the identified issues, the Campbell Collaboration can enhance its contribution to evidence‐based practice and policy, ultimately benefiting researchers, practitioners, policymakers, and the broader public who rely on high‐quality, timely evidence synthesis. There are instances where protocols are published but full texts are not, which may impact the overall understanding of the Campbell systematic review publication process. Future studies could consider including reviews with published protocols but without full‐text publications to provide a more comprehensive picture of the Campbell systematic review publication process. The Campbell Collaboration considers tracking and reporting on review projects that published protocols but were not ultimately completed, to provide a more comprehensive understanding of review completion rates and potential barriers. Given the potential importance of team experience, we strongly recommend future studies collect and analyze data in this area. This could include quantitative metrics such as the number of systematic reviews previously completed by team members, team members' academic backgrounds, and years of experience in relevant fields. Developing a standardized tool to assess team experience levels would facilitate more accurate comparisons across studies. Although this study couldn't directly analyze the impact of team experience, the potential importance of this factor suggests that team composition and experience levels should be considered when planning and managing Campbell systematic reviews. Providing additional training and support for novice teams, or incorporating experienced members into teams, may be effective strategies for improving efficiency.

**Figure 4:**
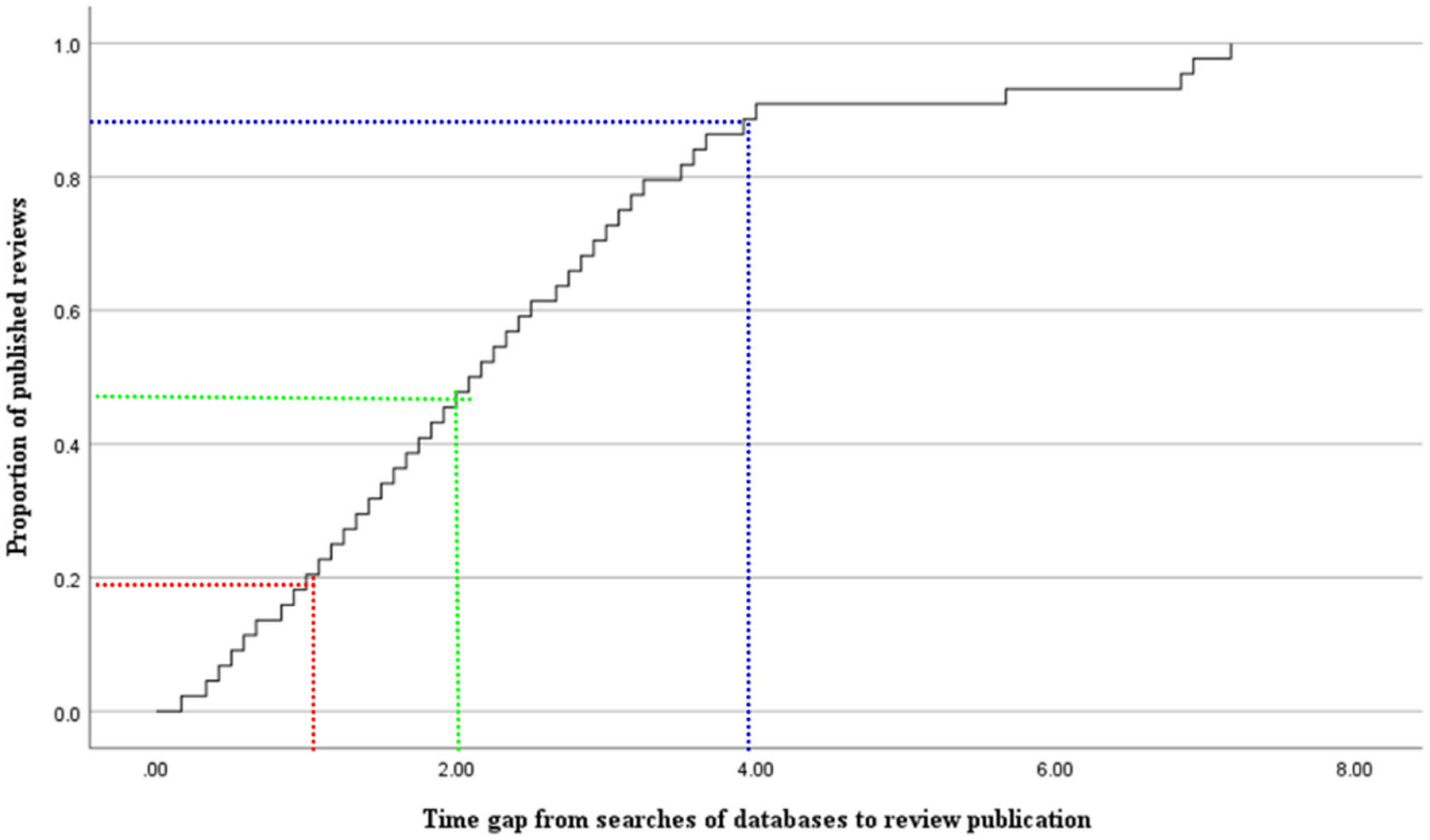
A Kaplan–Meier curve presenting the time gap from searches of databases to publication of the Campbell review.

Funding sources and associated timelines also can influence review completion times, especially in relation to Evidence and Gap Maps (EGMs), which often have tighter deadlines due to funder requirements. Future studies could propose a more systematic approach to exploring these factors, potentially through: (a) A survey of review authors to gather information on these aspects; (b) Case studies of reviews with particularly short or long completion times; (c) Analysis of any available metadata on review teams, funding, and scope (Figure 4).

## 7 CONCLUSION

This study reveals significant findings regarding the publication timelines of Campbell systematic reviews. Notably, half of Campbell reviews were published more than 2 years after their protocol registration, with substantial variations in median publication times across different review types. These results underscore the pressing need for optimizing the writing and publishing process of Campbell reviews. In conclusion, this study provides valuable insights into the current state of Campbell review publication timelines and offers a foundation for developing targeted strategies to optimize the review process. By addressing the identified issues, the Campbell Collaboration can enhance its contribution to evidence‐based practice and policy, ultimately benefiting researchers, practitioners, policymakers, and the broader public who rely on high‐quality, timely evidence synthesis (Table 3).

**Table 3:** Publication time from searches of databases to review publication.

Characteristics	Publication time in months, median (range)
**Year of review publication**	
2003–2006	34 (11–48)
2007–2010	22 (7–82)
2011–2014	18 (6–48)
2015–2018	19.5 (5–84)
2019–2023	20 (8–83)
**Campbell coordinating group**	
Aging	NA
Business and management	NA
Children and young person	20
Climate solutions	NA
Crime and justice	0
Disability	25.5
Education	20 (8–48)
International development	20 (7–83)
Knowledge translation and implementation	NA
Methods	37
Social Welfare	20 (5–48)
**Type of review**	
Systematic review	22 (2–83)
Evidence and gap maps	20 (11–36)
Methods research paper	37

NA, not available.

## PLANS FOR UPDATING THIS REVIEW

This review will be updated every 2 years.

## AUTHOR CONTRIBUTIONS

*Content*: Bei Pan, Long Ge, and Kehu Yang. *Systematic review methods*: Bei Pan, Long Ge, Kehu Yang, Xiaoman Wan, Ning Ma, and Zhipeng Wei. *Statistical analysis*: Bei Pan, Honghao Lai, and Liangying Hou. *Information retrieval*: Bei Pan, Long Ge, and Zhipeng Wei.

## CONFLICT OF INTEREST STATEMENT

The authors declare no conflicts of interest.
